# Long non-coding RNA NBAT1, TUG1, miRNA-335, and miRNA-21 as potential biomarkers for acute ischemic stroke and their possible correlation to thyroid hormones

**DOI:** 10.3389/fmolb.2022.914506

**Published:** 2022-09-30

**Authors:** Asmaa Mohammed, Olfat G. Shaker, Mahmoud A. F. Khalil, Mohammed Gomaa, Shaimaa A. Fathy, Abeer K. Abu-El-Azayem, Amira Samy, Mahmoud I. Aboelnor, Mohamed S. Gomaa, Othman M. Zaki, Randa Erfan

**Affiliations:** ^1^ Department of Medical Biochemistry and Molecular Biology, Faculty of Medicine, Fayoum University, Fayoum, Egypt; ^2^ Department of Medical Biochemistry and Molecular Biology, Faculty of Medicine, Cairo University, Cairo, Egypt; ^3^ Department of Microbiology and Immunology, Faculty of Pharmacy, Fayoum University, Fayoum, Egypt; ^4^ Department of Neurology, Faculty of Medicine, Fayoum University, Fayoum, Egypt; ^5^ Department of Internal Medicine and Endocrinology, Faculty of Medicine, Cairo University, Cairo, Egypt; ^6^ Department of Medical Microbiology and Immunology, Microbiology, Faculty of Medicine, Cairo University, Cairo, Egypt; ^7^ Department of Clinical and Chemical Pathology, Faculty of Medicine, Cairo University, Cairo, Egypt; ^8^ Department of Radiology, Faculty of Medicine, Fayoum University, Fayoum, Egypt; ^9^ Department of General Medicine, Faculty of Medicine, Fayoum University, Fayoum, Egypt; ^10^ Department of Clinical Pathology, Faculty of Medicine, Damietta University, Damietta, Egypt

**Keywords:** long non coding RNA, NBAT1, TUG1, miRNA-335, miRNA-21, ischemic stroke

## Abstract

**Objective:** RNA-based mechanisms of epigenetic modification related to acute ischemic stroke (AIS) have been widely studied recently. The current work aimed to determine the potential roles of four ncRNAs (TUG1 and its target miR-21, NBAT1, and miR-335) as promising diagnostic biomarkers in AIS as well as their involvement in the disease pathogenesis.

**Methods:** The levels of the studied lncRNAs and miRNAs were measured in the serum for two different groups, including patients with AIS (60) and healthy controls (60). All individuals were subjected to a full history investigation and clinical examination. Blood samples were tested for FBS, 2HPP, TAG, HDL, LDL, TSH, T3, and T4 levels.

**Results:** The serum levels of TUG1 were significantly increased in AIS patients compared to control subjects. It is worthwhile to note that serum TUG1 levels were positively correlated with cholesterol, triglycerides, LDL, carotid IMT (Intima-media thickness), and miR-21, while they were negatively correlated with HDL levels. Our study showed that NBAT1 serum expression levels were elevated in AIS patients compared to controls. NBAT1 expression levels were observed to be positively correlated with triglycerides, TUG1, and miR-21. NBAT1 could distinguish between AIS patients and controls with a sensitivity of 100% and specificity of 100% at a cut-off point of 1.45. Regarding miR-335, we found that its expression levels were downregulated in AIS patients compared with healthy controls. It could distinguish between AIS patients and controls with a sensitivity of 73.3% and a specificity of 100% at a cut-off point of 0.796.

**Conclusion:** Our results revealed that serum TUG1, miR-21, NBAT1, and miR-335 could be promising molecular diagnostic markers for AIS as these biomarkers could discriminate between AIS patients and healthy controls.

## Introduction

Ischemic stroke (IS) is one of the leading causes of disability and the second leading cause of death globally, after ischemic heart disease (Benjamin et al., 2019). IS is caused by an embolus or thrombus occluding a cerebral artery, reducing oxygen and blood flow to the brain. When this happens repeatedly, it leads to irreparable brain damage because of neuronal cell death. Another typical hallmark of IS is increased oxidative stress and inflammation (Chehaibi et al., 2016). The most common modifiable risk factors for stroke include diabetes mellitus and hypertension. Both can cause dyslipidemia and atherosclerosis (Johansson, 1999; Tuomilehto and Rastenyte, 1999). Another mechanism for hyperglycemia-related stroke risk is the increased brain lactate production and hypoperfusion in the infarction area. Hyperglycemia (blood sugar levels above 140 mg/dl) also reduces early blood flow restoration (Cai et al., 2020). Similarly, thyroid hormones also vary in the serum of patients with acute stroke (Marouli et al., 2020).

In addition to modifiable risk factors, increasing evidence suggests that epigenetics contributes to the etiology of cardiovascular diseases, specifically stroke susceptibility (Morgado-Pascual et al., 2018). MicroRNAs (miRNAs) and long non-coding RNAs (lncRNAs) are two RNA-based epigenetic regulatory mechanisms that are particularly noteworthy. Recently, lncRNAs (>200 nucleotides) have gained increased interest. LncRNAs can use both transcriptional and post-transcriptional pathways to control the expression of target genes (Chen et al., 2018). LncRNAs are involved in regulating the pathological process of ischemic stroke by altering atherosclerosis, inflammation, cell survival, and angiogenesis (Dykstra-Aiello et al., 2016).

MiRNAs are small single-stranded non-coding RNAs of 18–22 nucleotides. MiRNAs have been linked to neuronal development, synaptic plasticity, differentiation, metabolism, proliferation, and neurodegenerative disorders (Bartel, 2009). Nevertheless, several clinical diseases that contribute to IS, such as atherosclerosis, dyslipidemia, and inflammation, have been linked to changes in miRNA levels (Rink and Khanna, 2011). Furthermore, changes in several phases of stroke had different miRNA levels, implying that they could be used for diagnostic, therapeutic, and prognostic purposes (Mirzaei et al., 2018).

LncRNA taurine up-regulated gene 1 (TUG1) is a 7.1-kb lncRNA up-regulated by taurine (Khalil et al., 2009). TUG1 plays a role in developing non-small cell lung cancer, osteosarcoma, and bladder cancer (Zhang et al., 2013; Zhang et al., 2014; Liu et al., 2017). It is also linked to the development of atherosclerosis through modulating the miR-21/phosphatase and tensin homolog (PTEN) axis (Li et al., 2018). That is why both TUG1 and miR-21 can be implicated in AIS.

LncRNA Neuroblastoma-associated transcript1 (NBAT1) is a lncRNA that plays a role in carcinogenesis, and earlier reports confirmed NBAT1 dysregulation in various types of cancer (Yan et al., 2017). Polycomb repressive complex 2 (PRC2) signaling is one of the routes through which NBAT1 promotes the genesis and growth of tumors (Hu et al., 2015). It is worth mentioning that NBAT1 functional interaction with EZH2 (Enhancer of Zeste Homolog 2), a member of the PRC2 complex, plays a critical role in neurogenesis (Pandey et al., 2014) and causes suppressed expressions of its target genes, which are implicated in cell proliferation and cell migration (Pandey and Kanduri, 2015). Besides, NBAT1 can regulate gene expression of DKK-1 (Dickkopf-1) through modulating the functions of PRC2 as overexpression of NBAT1 represses EZH2 functions, which results in increased DKK1 levels (Pandey and Kanduri, 2015). Increased Dkk-1 expression is involved in the atherosclerosis process. High serum Dkk-1 levels have been consistently reported in patients with ischemic stroke (He et al., 2016). Moreover, Zhu et al. (2019), found that Dkk-1 may be a potential prognostic biomarker for ischemic stroke.

Recent research has related miR-335 to neural growth and development. Si and co-workers discovered a decrease in miR-335 expression in the adult rat brain after acute middle cerebral artery occlusion, leading to AIS-enhanced apoptosis (Si et al., 2019). On the other hand, the function of miR-335 in humans is uncertain. The current study target is to estimate levels of four ncRNAs (TUG1 and its target miR-21, NBAT1, and miR-335) in AIS patients. Additionally, this study investigated the possibility of utilizing these ncRNAs as diagnostic and prognostic biomarkers in AIS patients.

## Materials and methods

The current study protocol was authorized by Fayoum University Hospital’s local ethics committee. All procedures were carried out according to the Declaration of Helsinki. This report was performed following the Strengthening the Reporting of Observational Studies in Epidemiology (STROBE) principles (Moher et al., 2012).

### Study design and patients

This is a case-control study of 60 Egyptian individuals diagnosed with AIS within the first 24 h. Patients were chosen from the Neurology Departments at Fayoum University Hospitals. Patients were diagnosed using World Health Organization (WHO) guidelines, and symptoms appeared within 24 h (Hatano, 1976). Patients with a history of ischemic stroke, epilepsy, intracerebral hemorrhage, chronic kidney diseases, tumors, neurological diseases, and liver diseases were excluded. Moreover, patients with congestive heart failure, renal insufficiency, malignant tumors, severe edema, systemic infections, febrile disorders, recent surgery or trauma within the last 2 months, and autoimmune diseases were excluded. As a control group, 60 participants were involved in this study. They were similar in age, sex, and traditional vascular risk factors to the patients’ group. They sought medical assistance for a headache or spondylosis at the neurology clinic.

### Procedure of clinical examination

The participants were examined regarding their medical history, a general and comprehensive neurological examination, and a score on the National Institute of Health Stroke Scale (NIHSS) ranging from 0 to 42, with higher values indicating more severe neurologic damage (Brott et al., 1989). To confirm the diagnosis, all patients had magnetic resonance imaging (MRI) with a diffusion scan (stroke protocol) of the brain at Fayoum University Hospital’s Radiology Department.

### Carotid duplex examination

All the patients had their carotid arteries sonographically examined. They were positioned in a supine or semi-supine position, with their heads somewhat hyperextended and rotated 45° away from the side targeted by the exam. A lower frequency linear transducer (7 Megahertz) was used for the Doppler exam, while a higher frequency linear transducer (>7 Megahertz) was used to assess intima-media thickness and plaque morphology. A greyscale picture was used to evaluate intima-media thickness (carotid IMT) at the far wall of the common carotid artery, bulb, and internal carotid artery. The measurement included the media (echo-poor layer) and intima (echogenic layer). A thickness less than 1 mm in the intima-media was considered normal.

### Data collection and laboratory investigation

Erythrocyte sedimentation rate (ESR), serum C-reactive protein (CRP) level, Complete blood count (CBC), and liver and kidney function test to rule out the fasting blood glucose level, presence of metabolic or systemic disorders, and finally lipid profile were all performed as part of routine laboratory investigations.

TUG1, miR-21, NBAT1, and miR-335 were examined for their relative expressions. Each subject’s blood sample was collected (10 ml) using a vacutainer apparatus. The collected blood samples were placed in tubes with separator gels stuck between the serum layer (top) and the packed cells and were left to clot for 15 min before centrifuging at 4,000 ×g for 10 min. The serum was extracted from clotted whole blood and stored at −80°C until it was employed in the RNA extraction process (Bush et al., 2001).

### RNA extraction

A total sample volume of 100 μl serum was used for RNA extraction, and the extraction was carried out utilizing a miRNeasy extraction kit (Qiagen, Valencia, CA, United States). First, 500 ml QIAzol lysis reagent was added to the reaction mixture and incubated for 5 min at room temperature. Then, chloroform (100 μl) was added; the mixture was vortexed for 15 s and incubated for 2–3 min at room temperature. Then, centrifugation was done at 12,000 ×g at 4°C for 15 min. After removing the top aqueous phase, 1.5 times its volume of 100% ethanol was added. Then, we centrifuged each 700 μl of this mixture at 8,000 ×g for 15 s at room temperature on an RNeasy Mini spin column in a 2 ml collection tube. RW1 buffer (700 μl) was added to each column after the mixture had entirely passed and centrifuged at 8,000 ×g for 15 s at room temperature. The column was then filled with 500 μl buffer RPE and centrifuged at 8,000 ×g for 15 s at room temperature. Then, another 500 μl of buffer RPE was added to the column and centrifuged at room temperature for 2 min at 8,000 ×g. the column was placed in a new 1.5 ml collection tube and centrifuged for 2 min at 8,000 ×g. Finally, we pipetted 50 μl RNase-free water straight onto the column, followed by centrifugation for 1 min at 8,000 ×g to elute the RNA. The sample was treated with DNase after extraction to remove any remaining DNA before being reverse transcribed into cDNA with the DNase Max Kit (Qiagen, Valencia, CA, United States). Then, a NanoDrop 2000 spectrophotometer (Thermo Scientific, Waltham, MA, United States) measured the RNA at 260/280 nm.

### Reverse transcription

A high-capacity cDNA reverse transcription kit (Applied Biosystems, Foster City, CA, United States) was used to reverse transcript 1 μg RNA in a 10 μl final reaction volume, according to the manufacturer’s instructions (incubated for 60 min at 37°C, for 5 min at 95°C, and then maintained at 4°C).

### Noncoding RNAs expression by real-time quantitative PCR

QPCR primers and the miScript SYBR Green PCR kit (Qiagen) were used for performing the Quantitative PCR (qPCR). The levels of miR-335 and miR-21 gene expression were examined using SNORD 68 as an internal control (Ayeldeen et al., 2018). GAPDH, which was frequently employed as an internal control for serum lncRNAs in various studies, was used as an endogenous control for analyzing TUG1 and NBAT1 per the manufacturer’s method (Shaker et al., 2017). Table 1 lists the primer sequences utilized for each of the genes investigated. The PCR cycling condition protocol was performed as follows: 95°C for 10 min, then 40 cycles of 15 s at 95°C and 60 s at 60°C. Using the Rotor gene Q System, the operation was carried out on a 20 μl reaction mixture (Qiagen). Target genes were quantified concerning their endogenous control using the cycle threshold (Ct) approach. By subtracting the Ct values of SNORD 68 from miR-335 and miR-21, the ΔCt of microRNAs was computed. ΔCt of lncRNAs was estimated by subtracting GAPDH Ct values from TUG1 and NBAT1 Ct values.

**TABLE 1:** The primer sequences used for qRT-PCR.

Gene name	Forward primer	Reverse primer
NBAT1	5′-ACT​GAA​ACC​CAC​AGA​GAT​GAA​G-3^’^	5′-CCC​GTC​ATG​TAG​AGC​AAT​ATC​C-3^’^
TUG1	5′-CTG​AAG​AAA​GGC​AAC​ATC-3^’^	5′-GTA​GGC​TAC​TAC​AGG​ATT​TG-3^’^
miRNA-21	5′-ACA​CTC​CAG​CTG​GGT​AGC​TTA​TCA​GAC​TGA-3^’^	5′-CTC​AAC​TGG​TGT​CGT​GGA​GTC​GGC​AAT​TCA​GTT​GAG​TCA​ACA​TC-3^’^
miRNA-335	5′-AACTC​GAGTTC​AGC​CTT​CAT​TGT​TTA​ATC​TTT​ACA​ACA​GC-3^’^	5′-AAGAT​ATCTGT​ATG​GAC​ATG​AAG​CTT​TTA​CTT​CAA​CAT​TAG-3^’^
GAPDH	5′-CCC​TTC​ATT​GAC​CTC​AAC​TA-3^’^	5′-TGG​AAG​ATG​GTG​ATG​GGA​TT-3^’^

The equation ^2−ΔΔ^Ct was utilized to calculate the expression levels of miR-335, miR-21, NBAT1, and TUG1 (Livak and Schmittgen, 2001). The fold change values for the control group were set to 1. A negative or down-regulation is indicated by a fold change value less than 1, while a positive or up-regulation is indicated by a fold change value greater than 1 (Santoro et al., 2016). The four biomarkers’ fold changes were studied, and their diagnostic utilities in AIS patients were the primary parameters. The relationship between fold changes in the examined biomarkers and clinical disease activity and clinical presentation was one of the secondary outcomes.

### Statistical analysis

All data were collected and analyzed using SPSS version 22 (SPSS Inc., Chicago, IL, United States). The Chi-square test (for two or more groups) was used to analyze qualitative data, and the results were reported as numbers and percentages. An independent *t*-test (for two independent groups) and a one-way ANOVA (for two or more independent groups) were used to produce quantitative data in the form of standard deviation. Each research group’s quantitative data were tested for normality using the One-Sample Kolmogorov-Smirnov test, followed by inferential statistical analyses. The link between the variables was determined using a bivariate Pearson correlation test. The specificity and sensitivity of the studied variable were determined using a ROC curve analysis. P*-*Values of less than 0.05 were statistically significant.

## Results

### Demographic and clinical comorbidities of study groups

The clinical and demographic characteristics of study groups are illustrated in Table 2. With a *p*-value of >0.05, the table revealed no significant difference in medical history, age, or sex between groups. There was a significantly higher triacylglycerides (TAG) mean and a lower HDL mean among cases with a *p*-value <0.001. The mean carotid IMT among cases was (0.879 ± 0.24), and the mean NIHSS score was (12.88 ± 4.6). For the thyroid profile, the mean TSH was 4.47 ± 5.7 uI U/ml, with a mean free T3 of 2.62 ± 1.31 pg/ml and a mean free T4 of 1.84 ± 0.36 ng/dl.

**TABLE 2:** Comparisons of the demographic characteristics of the study groups.

Variables	Cases (*N* = 60)		Control (*N* = 60)		*p*-value
Age (years)					
Mean/SD	61.9	6.9	59.2	12.2	0.1
Sex					
Female	27	45%	36	60%	0.1
Male	33	55%	24	40%	
DM					
Yes	23	38.3%	20	33.3%	0.7
No	37	61.7%	40	66.7%	
HTN					
Yes	35	58.3%	36	60%	0.9
No	25	41.7%	24	40%	
Smoking					
Yes	31	51.7%	30	50%	0.9
No	29	48.3%	30	50%	
Lipid profile					
CHOL	161.6	45.8	158	28.4	0.6
TAG	128.8	62.3	73.4	33.5	<0.001**
LDL	102.6	39.7	91.6	28.5	0.08
HDL	34.8	9.3	53.9	13.4	<0.001**
Stroke severity					
Carotid IMT	0.879	0.24	—	—	—
NIHSS score	12.88	4.6	—	—	—
Thyroid profile					
TSH (uIU/ml))	4.47	5.7	—	—	—
Free T3 (pg/ml)	2.62	1.31	—	—	—
Free T4 (ng/dl)	1.84	0.36			

DM, diabetes mellitus; HTN, hypertension; FBS, fasting blood sugar; 2HPP, 2 h postprandial; TG, triglycerides; LDL, low-density lipoprotein; HDL, high-density lipoprotein; **(*p* < 0.01); NIHSS score, national institutes of health stroke.

### Serum expression levels of TUG1, NBAT1, miRNA-335, and miRNA-21 among cases

**FIGURE 1:**
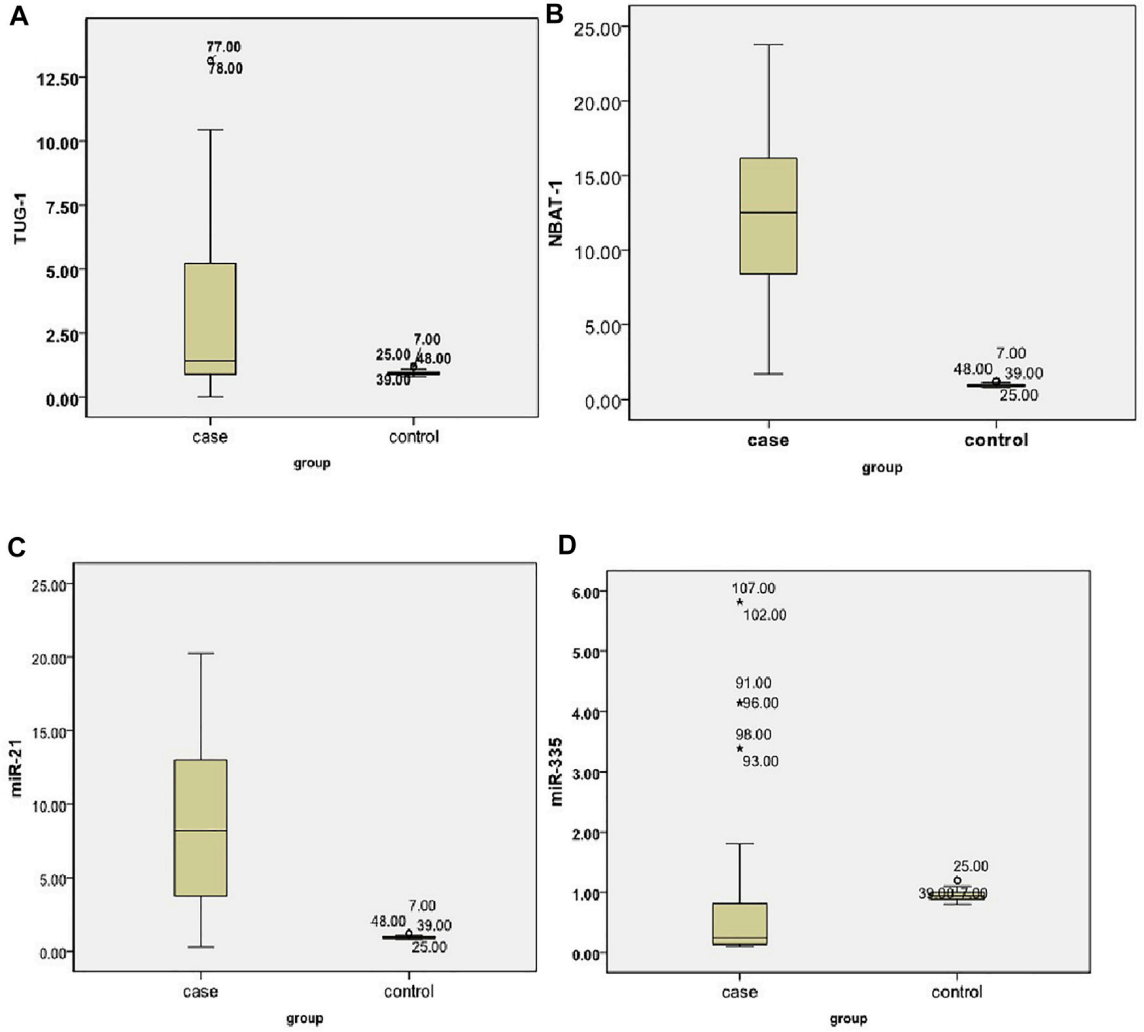
Expression levels of different biomarkers among cases **(A)** A dot plot for TUG1. **(B)** A dot plot for the NBAT1 levels in the study groups. **(C)** A dot plot for the miRNA-21 levels. **(D)** A dot plot for the miRNA-335 levels.

When compared to the control group, TUG1 and NBAT1 were strongly expressed in the serum samples of AIS patients. The fold change median of TUG1 was 1.42 in patients compared to controls with *p* < 0.001. The fold change median of NBAT1 was 12.5 in patients compared to controls with *p* < 0.001 (Table 3). Overexpressed miRNA-21 was demonstrated between the patients and control groups with a fold change median of 8.2. On the other hand, there was downregulation of the expression levels of miRNA-335 with a fold change median of 0.24 (Table 3) (Figures 1A–D).

**TABLE 3:** The expression levels of different markers in the study groups. There was a significantly higher median of TUG1, NBAT1, and miRNA-21 among cases and a lower fold change median of miRNA-335.

Variables	Cases (*N* = 60)		Control (*N* =60)		*p*-value
	Median	IQR	Median	IQR	
TUG1	1.42	4.3	0.945	0.12	<0.001**
NBAT1	12.53	8.04	0.945	0.12	<0.001**
miRNA-21	8.20	9.36	0.945	0.12	<0.001**
miRNA-335	0.245	0.67	0.945	0.12	<0.001**

** (*p* < 0.01).

### The association of different markers’ expression patterns and comorbidities among cases

Regarding the median of different biomarkers, no significant difference was observed between patients with comorbidities [diabetes mellitus (DM) and hypertension (HTN)], *p* > 0.05, as shown in Table 4.

**TABLE 4:** Comparisons of biomarkers in different comorbidities among cases.

Variables	TUG1	NBAT1	miRNA-21	miRNA-335
	Median/IQR	Median/IQR	Median/IQR	Median/IQR
DM				
Yes	1.14/5.9	10.9/10.9	8.2/7.8	0.15/1.6
No	1.43/3.5	14.2/7.8	7.6/10.6	0.41/0.64
*p*-value	0.9	0.8	0.3	0.2
HTN				
Yes	3.7/4.3	10.9/7.4	7.81/10.1	0.17/0.67
No	1.41/3.6	14.5/7.6	8.2/9.2	0.41/0.67
*p*-value	0.2	0.3	0.7	0.1

DM, diabetes mellitus; HTN, hypertension.

### Correlation between NIHSS score and study variables

**FIGURE 2:**
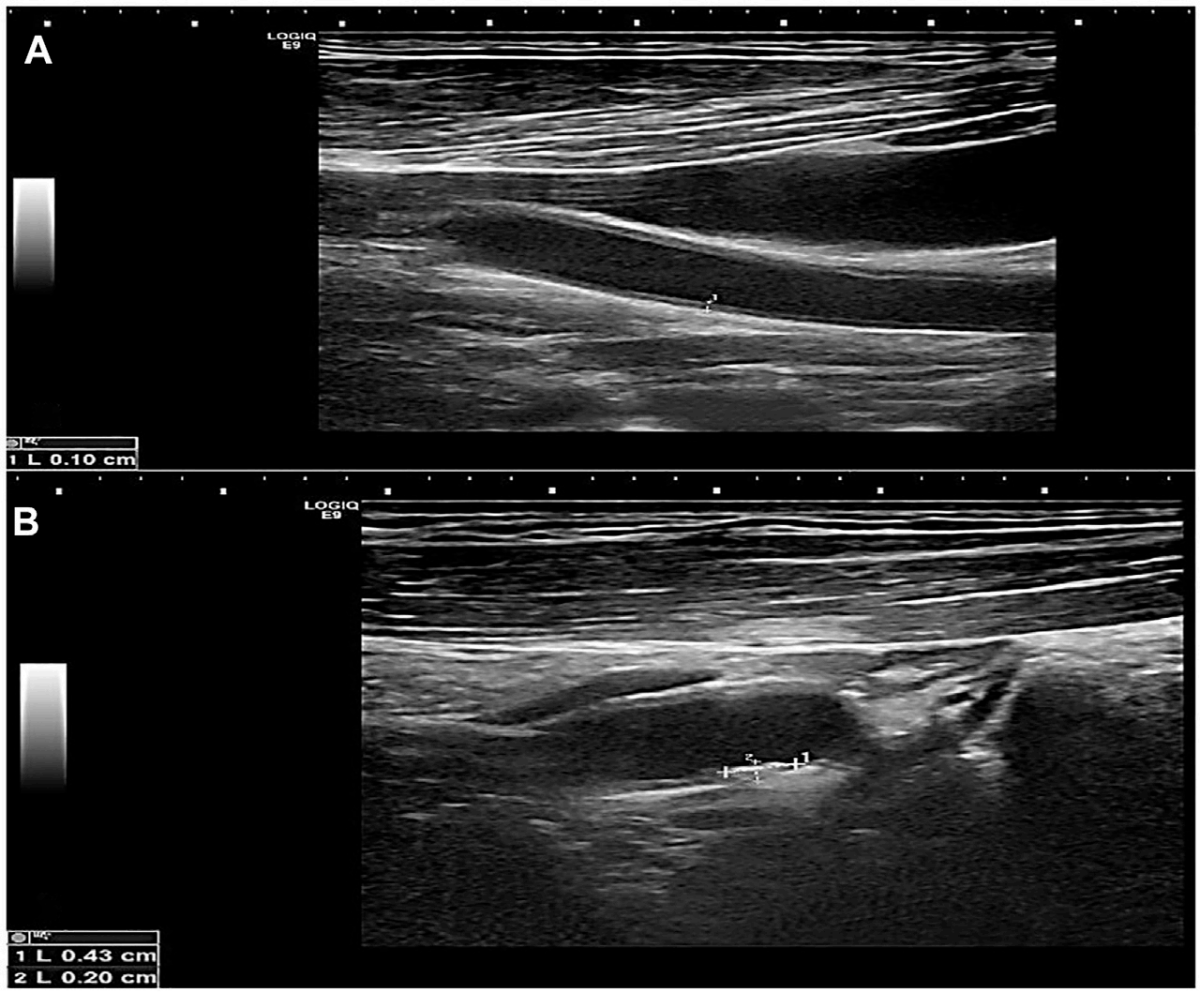
Greyscale longitudinal ultrasound images in a 60-year-old male diabetic patient showing **(A)** increased intimal/medial thickness at the left common carotid artery and **(B)** a calcified plaque at the proximal aspect of the left internal carotid artery.

**FIGURE 3:**
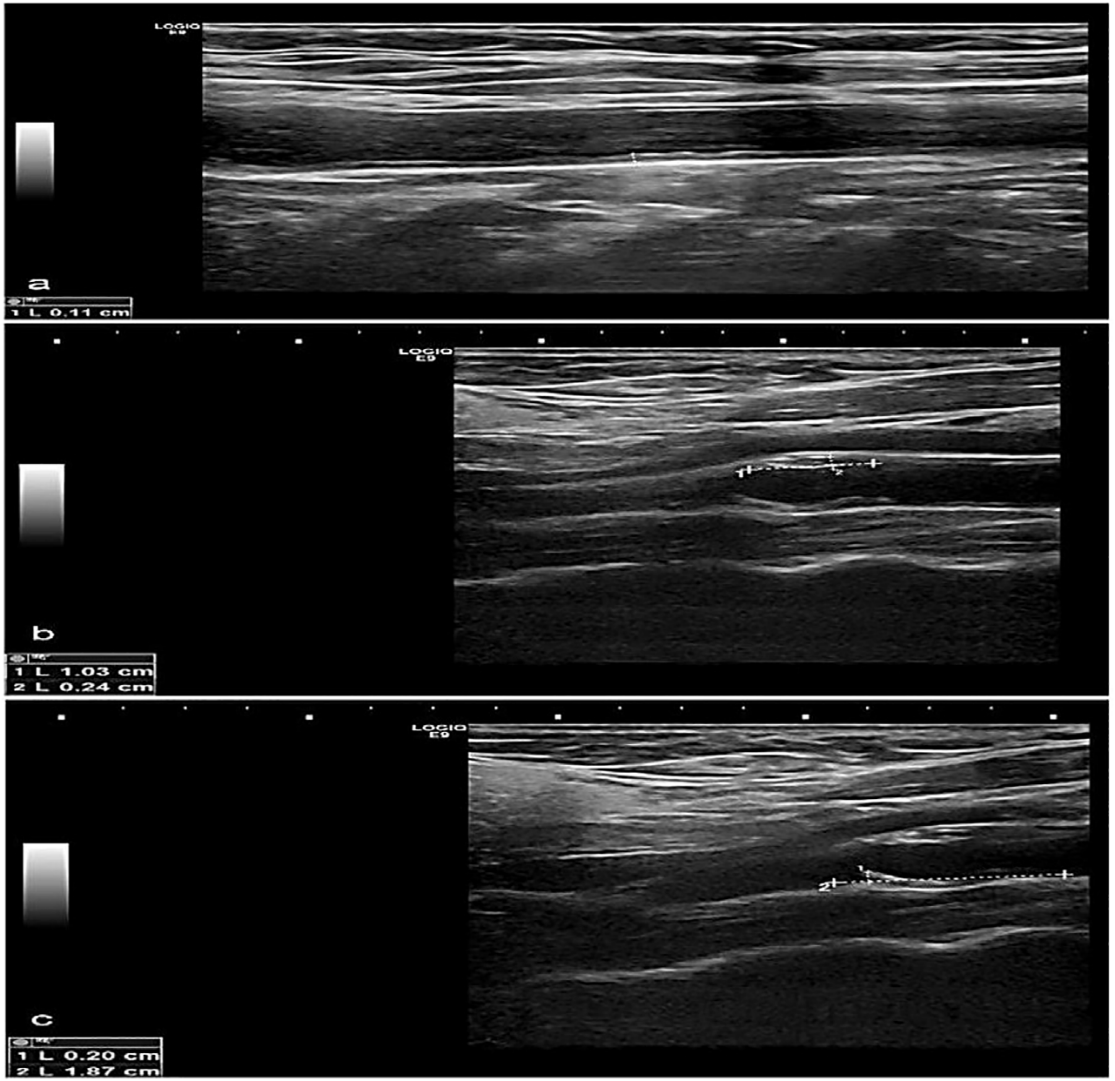
Greyscale longitudinal ultrasound images in a 70-year-old female diabetic and hypertensive patient showing **(A)** increased intimal/medial thickness at the right common carotid artery, **(B)** and **(C)** calcified plaques at opposing walls of the left carotid bulb.

There was a significant positive association between carotid IMT and NIHSS score with a *p*-value <0.05 (Figures 2, 3). Unlikely, we observed a negative correlation among cases with free T3 levels. Unlikely, no significant correlation was observed (*p*-value >0.05) in other thyroid profiles and biomarkers (Table 5).

**TABLE 5:** Correlation between NIHSS score and study variables among cases.

Variables	NIH score	
	r	(*p*-value)
Carotid IMT	0.32	0.01*
TSH (uIU/ml))	−0.12	0.4
Free T4 (ng/dl)	0.06	0.6
Free T3 (pg/ml)	−0.34	0.008**
TUG-1	0.17	0.2
NBAT-1	0.04	0.8
miRNA-21	−0.14	0.3
miRNA-335	−0.01	0.9

* (*p* < 0.05); ** (*p* < 0.01).

### Correlation between biomarkers and study variables

Serum TUG1 levels were positively correlated with cholesterol, triglyceride, LDL, free T4, and carotid IMT, in addition to NBAT1 and miRNA-21, while negatively correlated with HDL levels. Additionally, serum NBAT1 levels were positively correlated with triglycerides, TUG1, and miRNA-21, while negatively correlated with HDL levels. Considering miRNA-21, there was a positive correlation between it and TAG, TUG1, and NBAT1, while a negative correlation was found with HDL levels. Finally, a positive correlation was found between miRNA-335, carotid IMT, and TSH levels, while a negative correlation was found with cholesterol levels, as shown in Table 6.

**TABLE 6:** Correlation between biomarkers and study variables among cases.

Variables	TUG1	NBAT1	miRNA-21	miRNA-335
	r (*p*-value)	r (*p*-value)	r (*p*-value)	r (*p*-value)
CHOL	0.24 (0.01*)	0.01 (0.9)	0.08 (0.4)	−0.19 (0.03*)
TAG	0.37 (0.001**)	0.37 (0.001*)	0.26 (0.004**)	−0.12 (0.2)
LDL	0.21 (0.02*)	0.08 (0.4)	0.15 (0.1)	−0.17 (0.06)
HDL	−0.19 (0.03*)	−0.58 (0.001*)	−0.37 (0.001**)	−0.03 (0.8)
Carotid IMT	0.48 (0.001**)	−0.02 (0.9)	−0.29 (0.2)	0.27 (0.03*)
TSH (uIU/ml))	−0.09 (0.4)	0.11 (0.4)	−0.09 (0.5)	0.28 (0.02*)
Free T3 (pg/ml)	0.18 (0.2)	0.13 (0.3)	0.01 (0.9)	0.007 (0.9)
Free T4 (ng/dl)	0.35 (0.006**)	−0.12 (0.4)	−0.05 (0.7)	−0.25 (0.06)
TUG-1	—	0.27 (0.003**)	0.23 (0.01*)	−0.17 (0.06)
NBAT-1	—	—	0.56 (0.001**)	−0.02 (0.9)
miRNA-21	—	—	—	−0.07 (0.5)

* (*p* < 0.05); ** (*p* < 0.01).

### Evaluation of the diagnostic accuracy of TUG1, NBAT1, miRNA-21, and miRNA-335 serum levels

**FIGURE 4:**
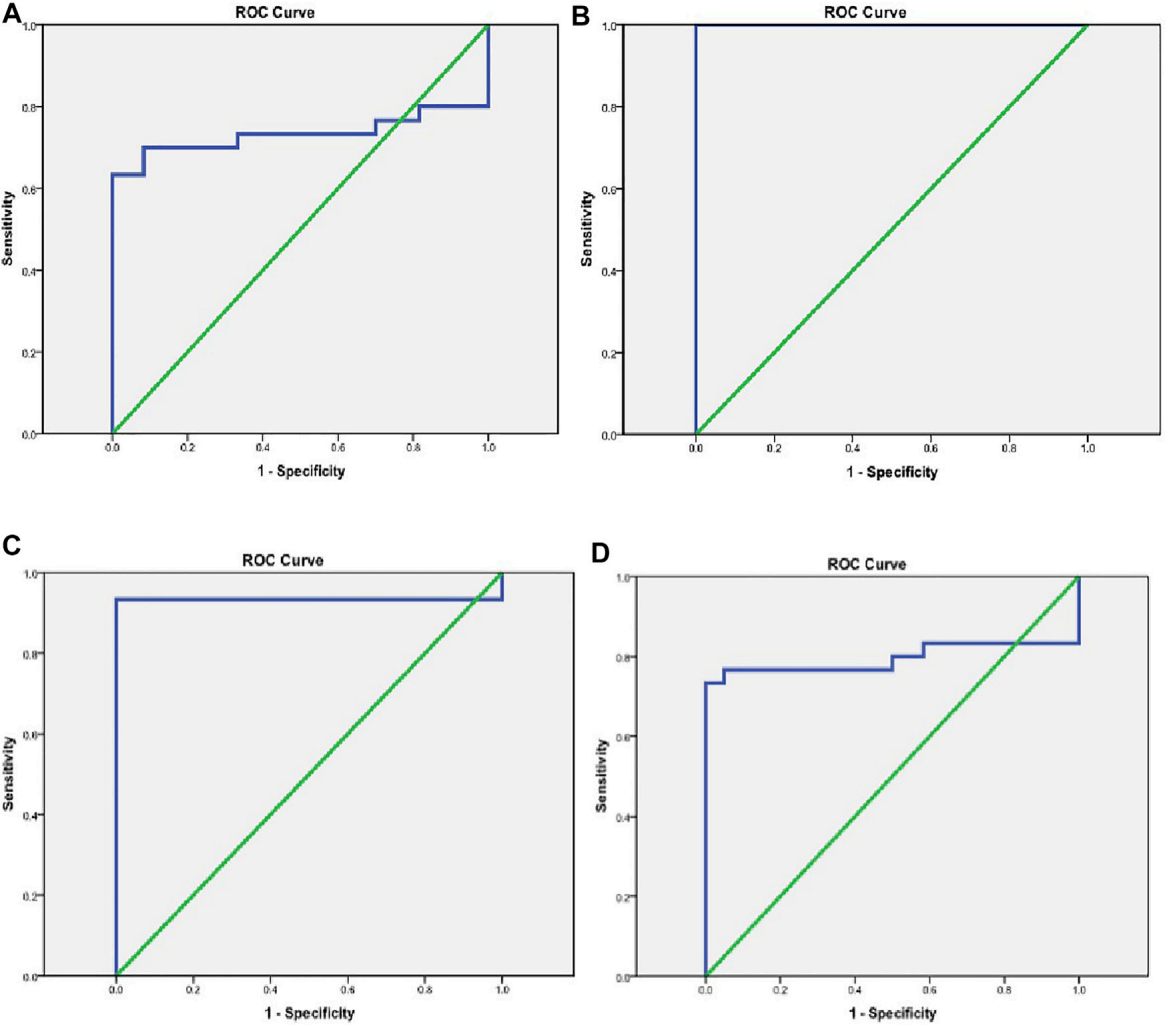
Evaluation of the diagnostic accuracy of TUG1, NBAT1, miRNA-21, and miRNA-335 serum levels. **(A)** A ROC curve for TUG1 in the diagnosis of AIS. **(B)** A ROC curve for NBAT1 in the diagnosis of AIS. **(C)** A ROC curve for miRNA-21 in the diagnosis of AIS. **(D)** A ROC curve for miRNA-335 in the diagnosis of AIS.

Biomarker tests in the diagnosis of AIS revealed good sensitivity and specificity in NBAT1, followed by miRNA-21 and miRNA-335 with a sensitivity of (100%, 93.3%, and 73.3%) and a specificity of (100% for all) at cut-off values of (1.45, 1.25, and 0.796), respectively. TUG1 showed high sensitivity (80%) and very low specificity (8.3%) (Table 7) (Figures 4A–D).

**TABLE 7:** Sensitivity and specificity of biomarkers in the diagnosis of AIS cases.

Variable	Sensitivity (%)	Specificity (%)	AUC (%)	Cut off point	*p*-value	CI
TUG1	80	8.3	73.3	0.853	<0.001**	0.627–0.838-
NBAT1	100	100	100	1.45	<0.001**	1–1
miRNA-21	93.3	100	93.3	1.25	<0.001**	0.87–0.99
miRNA-335	73.3	100	79.6	0.796	<0.001**	0.699–0.892

** (*p* < 0.01).

## Discussion

When cerebral blood flow is disrupted, oxygen and glucose availability to brain cells is reduced, resulting in AIS. Atherosclerosis in major intracranial arteries is one of the most common causes of stroke worldwide (Gorelick et al., 2008). The current study aimed to estimate the levels of four ncRNAs: TUG1 and its target miR-21, NBAT1, and miR-335 to determine their potential use as diagnostic and prognostic biomarkers in AIS patients and their possible relation to stroke-related risk factors and the patient’s thyroid profile.

TUG1 levels in the blood were substantially higher in AIS patients than in controls. TUG1 was also able to distinguish between AIS patients and control subjects, with a sensitivity of 80% and an AUC of 73.3%. It is noteworthy that serum TUG1 levels were positively correlated with cholesterol, TAG, LDL, carotid IMT, and miR-21 and negatively correlated with HDL levels. However, TUG1 was not significantly correlated with the NIHSS score of the patients and was not associated with comorbidities (DM or HTN).

This is consistent with previous work by Wang et al. (2019), who found that TUG1 microglia were involved in neuroinflammation following an ischemic stroke. TUG1 was increased in microglial cells and behaved as a sponge for miR-145a-5p. TUG1 silencing shifted the microglia phenotype (from M1 to M2) and reduced pro-inflammatory cytokines, promoting the production of anti-inflammatory cytokines, and improving cell survival. The deletion of TUG1 also inhibited the activation of the nuclear factor- κB (NF-κB) pathway produced by oxygen-glucose deprivation (OGD)/R. As a result, TUG1 is thought to synchronize microglia and inflammatory cytokine assembly immediately after an OGD insult. Besides, neuron apoptosis represents one of the primary causes of AIS in patients. Apoptosis in AIS can be influenced by both downregulation and upregulation of a specific ncRNA. TUG1 promotes apoptosis in neurons by sponging miR-9 and increasing Bcl2l11 expression (Chen et al., 2017). Moreover, the mentioned positive correlation between TUG1 and cholesterol, TAG, LDL, and carotid IMT confirmed the direct relation of this biomarker to atherosclerosis which is one of the most direct risk factors of AIS. Therefore, the current study agrees with the study of Zhang and co-workers, who detected that by regulating fibroblast growth factor1 (FGF1) *via* miR-133a, TUG1 knockdown suppressed hyperlipidemia (Zhang et al., 2018).

We found that miR-21 was a target gene of TUG1 by Starbase prediction. Moreover, our findings are consistent with Li et al. (2018), who found that TUG1 was more expressed in patients with atherosclerosis than in healthy volunteers. Their study proved that TUG1 expression level was reversely correlated with PTEN expression in patients with atherosclerosis through competing with PTEN for miR-21 binding. So, they considered that TUG1 could be a potential target for treating atherosclerosis. They also discovered that miR-21 expression was positively linked with the expression of TUG1 and that downregulating TUG1 significantly decreased miR-21 expression. Our results agree with the previously mentioned study, as upregulation of miR-21 was demonstrated in the patients, and we found a positive correlation between miR-21 and TUG1.

Furthermore, miR-21 and TAG positive association, in addition to miR-21 and HDL negative correlation, have been related to metabolic syndrome and implicated in the proliferation and development of human adipose tissue-derived mesenchymal stem cells (hASC) (Kim et al., 2012). A substantial correlation between plasma miR-21 and IMT was described by Fontanella et al. (2021) as a surrogate marker for early atherosclerosis. Chen et al. (2019) also discovered that angiotensin II-induced angiogenic sprouting in human microvascular endothelial cells (HMECs) was linked to the STAT3/miR-21 pathway. Their findings suggest that targeting the STAT3/miR-21 axis in conjunction with current atherosclerosis therapies could be effective. We conclude from the above that TUG1 and its target miR-21 have a possible relation to the pathogenesis of AIS and atherosclerosis which is one of the most important risk factors for AIS.

To the best of our knowledge, this is the first report to investigate the levels of NBAT1 in AIS patients. The levels of NBAT1 in AIS patients were higher than in controls. Also, serum NBAT1 levels were positively correlated with TAG, TUG1, and miR-21 while negatively correlated with HDL levels. Moreover, it was found that NBAT1 could distinguish between AIS patients and control subjects with a sensitivity of 100% and a specificity of 100% at a cut-off point of 1.45. The specific role of NBAT1 in AIS pathogenesis is unclear but it may act through regulation of DKK-1 as overexpression of NBAT1 represses EZH2 functions, which results in increased DKK-1 levels (Pandey and Kanduri, 2015). Increased Dkk-1 expression is involved in the atherosclerosis process. High serum Dkk-1 levels have been consistently reported in patients with ischemic stroke (He et al., 2016). Moreover, Zhu et al. (2019), found that Dkk-1 may be a potential prognostic biomarker for ischemic stroke.

The present study demonstrated that miR-335 expression was lower in AIS patients than in control subjects. It could distinguish between AIS patients and controls with a sensitivity of 73.3 percent and a specificity of 100 percent at a cut-off point of 0.796. These findings agree with that of Si et al. (2019), who discovered that miR-335 was downregulated during AIS in rat models, related to decreased stress granules (SG) formation, elevated Rho-associated protein kinase 2 (ROCK2) expression, and higher apoptotic levels. They also discovered that treating mice with miR-335 increased SG formation, reduced ROCK2 protein expression and apoptosis, and reduced ischemia-induced infarction. Their findings showed that miR-335 prevented apoptosis and enhanced SG formation in AIS *via* lowering ROCK2 expression, suggesting that it could be a therapeutic target for brain injury. Besides, we found for the first time a positive correlation between miR-335 and carotid IMT.

Zhao et al. (2016) also found that plasma miR-335 levels were lower in AIS and negatively correlated with NIHSS scores, in contrast to plasma Calmodulin (CaM) levels. In patients who have never had intrinsic thyroid disease, changes in thyroid hormone concentrations are frequently related to critical illness. Non-thyroidal illness syndrome is the name for this condition (NTIS, or euthyroid sick syndrome). The most common hormone pattern in NTIS is T3 deficiency with normal thyroid-stimulating hormone (TSH) and thyroxine (T4) levels. The alterations in thyroid hormones can be due to NTIS or disturbances in the hypothalamic-pituitary-thyroid axis (Nagri et al., 2021).

In our study, we found a negative correlation between T3 and the NIHSS. This is agrees with Zhang and co-workers, who state that a low FT3 value on admission was associated with stroke severity, subtype, and prognosis (Zhang et al., 2019). Various miRNAs and lncRNAs clearly affected by thyroid hormones and receptors have been identified (Zhao et al., 2019). One of these lncRNAs is TUG1. Lin and co-workers found that T3/TR treatment reduced TUG1 expression *in vitro*, resulting in the downregulation of alfa fetoprotein (AFP) mRNA in patients with non-hepatitis B/non-hepatitis C HCC (NBNC-HCC) (Lin et al., 2020). Our results showed that TUG1 was positively correlated with T4 in the presence of acute stroke. To our knowledge, no previous study has investigated this correlation in this clinical setting.

Our work also found a positive correlation between miR-335 and TSH in AIS. Our study’s relatively small sample size is a limitation, necessitating additional investigations with bigger sample sizes. In conclusion, serum TUG1, miR-21, NBAT1, and miR-335 are promising molecular diagnostic markers for AIS. The correlation of TUG1 and miR-335 with T4 and TSH, respectively, in acute ischemic stroke opens a new channel to study how this correlation may play a role in severe acute illness.

## Data Availability

The original contributions presented in the study are included in the article/supplementary material, further inquiries can be directed to the corresponding authors.
